# Adipose Morphology: a Critical Factor in Regulation of Human Metabolic Diseases and Adipose Tissue Dysfunction

**DOI:** 10.1007/s11695-020-04983-6

**Published:** 2020-10-06

**Authors:** Fangcen Liu, Jielei He, Hongdong Wang, Dalong Zhu, Yan Bi

**Affiliations:** 1 Department of Endocrinology, Nanjing Drum Tower Hospital Clinical College, Nanjing Medical University, Nanjing, China; 2 Department of Endocrinology, Nanjing Drum Tower Hospital, The Affiliated Hospital of Nanjing University Medical School, Nanjing, China

**Keywords:** Adipocyte hypertrophy, Obesity, Insulin resistance, Adipose dysfunction

## Abstract

Emerging evidence highlights that dysfunction of adipose tissue contributes to impaired insulin sensitivity and systemic metabolic deterioration in obese state. Of note, adipocyte hypertrophy serves as a critical event which associates closely with adipose dysfunction. An increase in cell size exacerbates hypoxia and inflammation as well as excessive collagen deposition, finally leading to metabolic dysregulation. Specific mechanisms of adipocyte hypertrophy include dysregulated differentiation and maturation of preadipocytes, enlargement of lipid droplets, and abnormal adipocyte osmolarity sensors. Also, weight loss therapies exert profound influence on adipocyte size. Here, we summarize the critical role of adipocyte hypertrophy in the development of metabolic disturbances. Future studies are required to establish a standard criterion of size measurement to better clarify the impact of adipocyte hypertrophy on changes in metabolic homeostasis.

## Introduction

Obesity is a primary cause of metabolic disorders including type 2 diabetes (T2D), dyslipidemia, and hypertension. The prevalence of obesity has doubled in 73 countries all over the world and over 2 billion people are suffering from overweight and obesity [1]. Importantly, overnutrition predisposed individuals to insulin resistance and metabolic abnormalities [1, 2]. In obese individuals, white adipose tissue (WAT), which mainly mediates energy homeostasis, becomes dysfunctional and expands improperly to store excess energy. When lipid accumulation exceeds adipose maximum storage capacity, lipolysis increased, releasing fatty acid into circulation [1, 2]. Subsequently, ectopic fat deposits in peripheral organs such as the liver or skeletal muscles and eventually leads to abnormal glucose metabolism and systemic insulin resistance. This phenomenon has been commonly referred to as “lipotoxicity” [3, 4].

In the past few decades, fat mass content has been regarded as a key factor that influences the severity of metabolic disturbances. In recent years, numerous researches have focused on how the excessive energy is stored (hypertrophy or hyperplasia) in adipose tissue (AT) rather than the total fat mass content [5–7]. Physiologically, adipocytes expand or proliferate in order to store more energy as triglyceride. However, in morbid obesity, adipocytes tend to expand to the greatest extent, which is termed as “adipocyte hypertrophy”. Even within the same WAT depot, cell diameter of different adipocytes varies dramatically, ranging from less than 20 to 300 μm [8]. Accordingly, alteration in adipocyte size as well as the proportions of small or large adipocytes could manifest the development of T2D and non-alcoholic fatty liver disease (NAFLD) [9–12]. Importantly, enlargement of adipocytes triggers low-grade chronic inflammation, insufficient angiogenesis, and excessive collagen deposition, which further lead to abnormal adipokine release and impaired glucose metabolism [13].

In this review, we aim to outline a comprehensive overview of adipocyte hypertrophy in terms of metabolic disorders; we also summarize underlying mechanisms and discuss the role of adipocyte size as a potential marker in the treatment of obesity-related metabolic diseases.

## Distinction Among White Adipocyte Size in Different Depots

Generally, larger adipocytes are critically associated with metabolic disorders [14]; yet, the effect of enlarged adipocytes on metabolic dysregulation varied in different adipose depots.

AT is commonly classified as visceral AT (VAT) and subcutaneous AT (SAT). Anatomically, VAT is located primarily in the mesentery and omentum while SAT is presented mainly in gluteofemoral, back, and anterior abdominal region. Metabolically, SAT is suggested to have a protective role of metabolic risk [15, 16], while excessive VAT accumulation is an independent risk factor for obesity-induced metabolic disorders [17].

Specifically, VAT contains fewer preadipocytes and more large adipocytes. On the contrary, SAT tended to contain more small adipocytes [18]. One study measured adipocyte size in both SAT and VAT in 11 morbidly obese women with normal glucose level. Mean adipocyte volume was larger in VAT than that in SAT, but these two depots did not differ in the proportion of small adipocytes [14]. Also, expressions of cell differentiation markers were greater in SAT, whereas VAT displayed greater expression of inflammatory genes [19]. These results suggest that VAT tends to be less involved in triglyceride deposition but relates more to adipose inflammation, compared with SAT.

With respect to the relationship between adipocyte size and lipid/glucose profile, some studies indicated that increased visceral adipocyte size (VAS) had a much stronger detrimental influence on plasma total cholesterol, LDL cholesterol, apolipoprotein B, and triacylglycerols. On the other hand, subjects with greater subcutaneous adipocyte size (SAS) displayed higher homeostasis model assessment of insulin resistance (HOMA-IR**)**, blood glucose level, and plasma insulin level [12, 20, 21]. This distinction could be explained by different blood supply in VAT and SAT depots, since VAT is mainly drained by portal vein into the liver where lipoprotein and lipids are synthesized [22].

## Distinction Among White Adipocyte Size in Different Genders

There are strong differences in SAT and VAT biology in male and female subjects. Sex differences in fat distribution involve both cell size and number. Traditional view suggested that gluteo-femoral adipocytes of women are larger while abdominal adipocytes are comparable between both genders, and visceral adipocytes of women are significantly smaller [23]. Recent research indicated that males have larger adipocytes in both SAT and VAT than females independent of the content of adiposity [24]. Generally, at lean state, premenopausal women have a greater amount of small adipocytes especially in the femoral depot [25, 26]. Also, as lipid accumulating, men have more hypertrophic type of adipose expansion compared with women, suggesting a gender difference in adipose tissue remodeling and fatty acid storage [27].

One potential contributor to sex differences in adipose tissue expansion is the number of adipocyte precursor cells within adipose tissue. Previous animal studies showed that compared with male mice, female C57BL/6J mice have more adipocyte precursor cells (APC) with low-fat diet and increased more APCs with high-fat diet in VAT (gonadal adipose tissue (GWAT)) and SAT (inguinal white adipose tissue (IWAT)) [28]. These differences may explain why males display a higher degree of insulin resistance although inflammatory markers in GWAT were similar [28].

Accordingly, different patterns of adipocyte proliferation were attributed to sex hormones and receptors. It has been proved that estrogens influence hyperplasia by increasing APCs number and stimulating cell proliferation [29, 30]. Estrogen receptor alpha (ERα) and estrogen receptor beta (ERβ) are estrogen receptors on adipocytes which influence adiposity [27]. The total body ERα knockout mouse has increased adiposity, increased visceral fat accumulation, and the metabolic syndrome. Males have a relative lack of ERα in the visceral depot and are therefore primed to store more fat viscerally [31]. Future investigations are required to better clarify the effect of sex hormones on systemic adipose biology.

## Relationship Between Adipocyte Size and Metabolic Disorders

### Obesity and Insulin Resistance

Of note, during the acceleration of adipose content, dysfunctional AT leads to defects in insulin sensitivity and the development of insulin resistance. Most studies suggested that enlarged SAS has been implicated in the progression of impaired regional and systemic insulin sensitivity [5, 9, 32–35]. This phenomenon could further counteract insulin-promoted adipogenesis.

In a group of non-diabetic individuals, SAS negatively correlated with insulin sensitivity measured by euglycemic-hyperinsulinemic clamp (M value) [5, 9]. In line with the association, similar studies reported compared with insulin-sensitive subjects, body mass index (BMI)-matched insulin-resistant overweight/obese subjects displayed larger SAS [32, 34, 35]. Also, in the field of VAT, VAS was found to be positively related to HOMA-IR in lean patients after adjustment of age and body mass [36, 37]. In contrast, one study indicated that no association was found between VAS and HOMA-IR in non-diabetic subjects [9]. Notably, mesenteric adipocyte size was suggested to be strongly related to insulin resistance compared with the other two visceral adipose depots [38]. Inconsistencies among prior studies may be explained by the different sample size, adjustment of different metabolic variables, and distinct adipose depots.

Despite abundant cross-sectional studies, some interventional studies explored the association of adipocyte size alterations during a short-term weight gain. In one recent approach, recruited metabolically healthy obese subjects were given a hypercaloric diet to induce 3.2-kg weight gain in a period of 4 weeks. At baseline, the insulin-sensitive (IS) subjects displayed smaller SAS. Surprisingly, compared with insulin resistance (IR) group, the proportion of small cells in IS group decreased from 50 to 43% while no significant alteration was observed in IR group [34]. This result indicated the negative impact of weight gain even in subjects who were metabolically healthy. Likewise, another overfeeding study which made 29 normal weight men gain 7.6 kg in 8 weeks suggested that subjects who had greater number of small adipocytes displayed greater decrease in insulin sensitivity [39].

These results highlighted “adipogenic potential”. Specifically, when enlargement of adipocytes reached maximal capacity for fat storage and recruitment and maturation of preadipocytes were significantly impaired, accumulation of small adipocytes and concomitant adipocyte hypertrophy occurred, leading to excess lipid deposition in ectopic tissues [40, 41]. It is noteworthy that compared with the study which recruited obese subjects [34], the latter study enrolled young, normal-weight men [39]. Moreover, the definition of “small adipocytes” was different between these two studies [34, 39]. In addition, these studies emphasized cell distribution along with the absolute value of adipocyte size.

### Type 2 Diabetes

The relationship between enlarged adipocyte size and deterioration of blood glucose level is well confirmed. SAS was higher in subjects with impaired glucose tolerance (IGT) and T2D than subjects with normal glucose tolerance (NGT) [5, 42, 43]. Average cell size was positively correlated with systemic glucose tolerance [5]. Consistent result was obtained in another study which showed an increasing trend of average SAS in lean, impaired fasting glucose (IFG) or IGT and T2D subjects [44]. In terms of specific cell distribution, more large adipocytes and less small adipocytes were observed in T2D patients, indicating that elevated blood glucose level is accompanied by recruitment and proliferation of adipocyte precursors, though the capability to develop mature adipocytes has been impaired [45].

Interestingly, most of the studies focused on abdominal SAT while some studies compared different SAT depots. Subcutaneous abdominal adipocyte size (AAS) was identified as a risk factor for the progression of T2D in middle-age women, whereas femoral adipocyte size (FAS) displayed weaker association with T2D only via the correlation with AAS [6]. This study highlighted the impact of SAT present in the abdominal area on normal glucose metabolism. In addition, an overfeeding study suggested upper-body adipocyte size increased significantly in response to overfeeding. However, lower-body adipocyte responded to excessive food intake by hyperplasia [46]. The inherent dynamics of preadipocytes may result in distinct characteristics of SAT from different depots.

With respect to visceral adipocyte size (VAS), much less is known about VAS alteration in T2D patients, most of the studies were similar to obese and insulin-resistant subjects. Few studies with small sample size demonstrated that type 2 diabetic patients presented greater VAS [36, 38, 42, 47]. It has been speculated that compared with SAT, the number of visceral adipocytes was notably more important than adipocyte size [21]. There is a need to better understand the relationship between VAS and hyperglycemia in future observations.

### NAFLD

NAFLD, one important component of metabolic syndrome (MS), is critically involved in the progression of T2D. One study highlighted that SAS could explain 21% of liver fat deposition according to linear regression [48]. In the field of VAT, liver injury was generally assessed by alanine aminotransferase (ALT), aspartate aminotransferase (AST), and NAFLD activity score. Increased omental adipocyte diameter associated tightly with ALT, AST level, and NAS, suggesting that omental adipocyte enlargement was an independent predictor of stages of NAFLD together with liver injury level [49, 50].

### Other Metabolic Diseases

Few studies found that women with polycystic ovary syndrome (PCOS) indicated enlarged SAS compared with healthy women matched for age and BMI levels [51, 52]. Similarly, VAS also increased in women with PCOS [53]. In addition, one study measured SAS in normal weight and obese pregnant women, where cell diameter larger than 150 μm was defined as “very large adipocytes”. Notably, HOMA-IR level in trimesters 3 was closely associated to proportion of very large adipocytes, which indicates the potential role of adipocyte size in predicting the development of gestational diabetes [54]. More investigations are required for further exploration of extensive change during pregnancy.

## Association Between Adipocyte Hypertrophy, Pathophysiological Changes, and Metabolic Dysfunction in AT

**Fig. 1:**
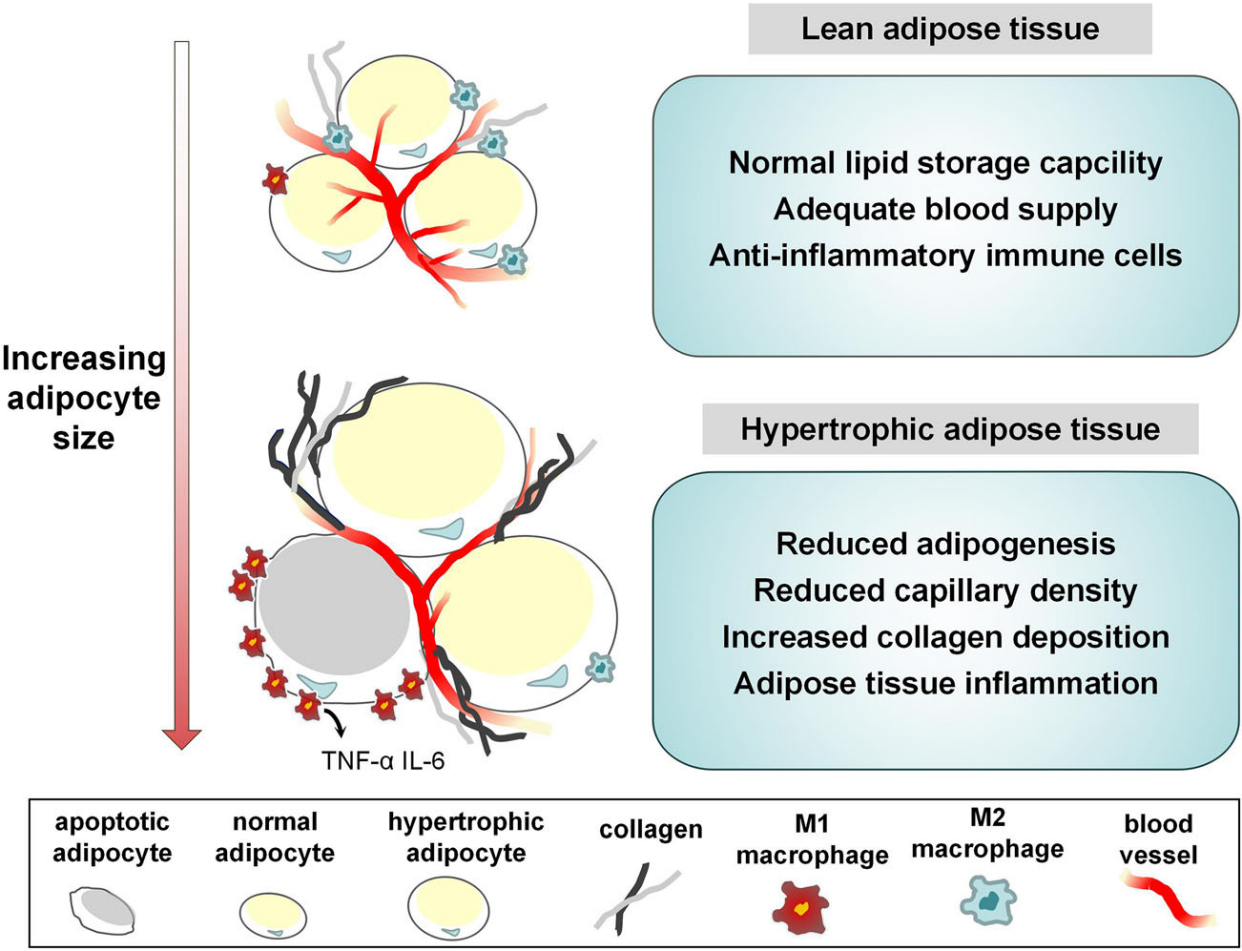
Adipocyte hypertrophy-induced and metabolic dysregulations. With the increase of lipid accumulation, adipocyte hypertrophy is associated with, exacerbation of inflammation, adipocyte hypoxia, excessive collagen deposition, abnormal adipokine release, and impaired glucose metabolism, which finally contribute to dysregulated systemic energy metabolism

Various pathological changes occur in AT following the enlargement of adipocyte size. On the one hand, adipocyte hypertrophy triggers inflammatory response and exacerbates regional hypoxia, further making excessive collagen deposition. On the other hand, pathophysiological changes subsequently contribute to dysregulation of adipokine release and impaired glucose metabolism. Adipocyte hypertrophy serves as an important factor which connects closely to metabolic dysfunction in AT (Fig. 1).

### Infiltration of Inflammatory Immune Cells

Apart from adipocytes, various types of cells including adipocyte progenitors, fibroblasts, endothelial cells, and immune cells were discovered in AT from healthy individuals [55–57]. With the development of obesity, a high-degree of inflammation, marked by elevated infiltration of pro-inflammatory immune cells, was discovered in WAT [58–61]. Among all the pro-inflammatory immune cells, M1 macrophages, which have been reported to infiltrate into WAT in both obese human and animals, produce proinflammatory cytokines such as tumor necrosis factor α (TNF-α), interleukin-6 (IL-6), and interleukin-1b (IL-1b) [62–66].

On the one side, hypertrophic adipocytes exacerbate infiltration of proinflammatory immune cells and secretion of inflammatory cytokines. In obese individuals, enlargement of adipocyte size associates closely with increased recruitment of M1 macrophages and reduced anti-inflammatory M2 macrophages [19, 67–70]. Interestingly, increased M1 macrophages were regarded as a protective compensatory mechanism to limit adipocyte hypertrophy since macrophages could promote collagen accumulation in obese state [71]. When chemokine CC receptor (CCR)2, the receptor of monocyte chemoattractant protein 1 (MCP-1), was knockout, the number of macrophages was decreased, associated with elevated adipocyte diameter [65, 72, 73]. Also, in SAT, induced natural killer T (iNKT) cells, which are one subset of T cells, were depleted in obesity. When iNKT cells were transferred into obese mice, body weight and adipocyte size were observed to decrease significantly [74]. Additionally, enlarged adipocytes overexpressed inflammatory cytokines such as NF-κB [74], IL-6 [75], MCP-1 [55, 61], and TNF-α [60, 76] independent of BMI level and total body fat mass content. Also, in obese subjects, plasma C-reactive protein (CRP) level was positively correlated to the proportion of hypertrophic adipocytes [76].

On the other side, inflammatory cytokines may contribute to enlargement of adipocyte size. It has been reported that TNF-α in SAT hindered the differentiation process of preadipocytes via suppression of mesenchymal stem cell (MSC) commitment towards adipogenic differentiation [77]. Further, TNF-α reduced activity of the transcription factor EBF transcription factor 1 (EBF1), thereby promoting the development of adipocyte hypertrophy through altered expressions of key lipolytic genes [13]. One study showed that M1 macrophages were positive correlation with VAS but not with SAS and in obese T2D and obese non-T2D subjects [36]. This correlation indicates that VAS has more pronounced inflammatory phenotype, although SAT accounts for about 80% of whole-body fat mass. Further studies are required to identify whether VAS associates closer with inflammation in lean subjects.

### Hypoxia and Angiogenesis

When AT undergoes insufficient oxygen supply, hypoxia-inducible factor-1α (HIF-1α) is activated. Subsequently, increased fiber deposition remodeling and proinflammatory phenotype are triggered, contributing to the progression of adipose dysfunction and insulin resistance [78, 79]. It has long been suggested that enlargement of adipocyte size limits oxygen diffusion and triggers regional hypoxia [78–81]. Generally, the hypoxic state of hypertrophic adipocyte could be ascribed to two factors. First, compared with small cells (< 66 μm), cells with large size (> 88 μm) consumed more oxygen [82]. Also, when diameters of hypertrophic adipocytes exceed 100 μm, the diffusion rate of oxygen declines profoundly [64, 83].

In recent decades, some studies suggested that the contribution of adipocyte hypertrophy to the hypoxia response becomes less important [84, 85]. One study found that in obese patients, only a small proportion of adipocytes diameter exceed 100 μm, suggesting that the hypoxic condition within hypertrophic adipocytes may also have been caused by other factors [86]. Notably, in obese state, AT blood flow is about 30–40% lower than subjects with normal weight [87]. It was noteworthy that glycation-induced inability of angiogenesis decreased blood flow [85]. Moreover, imbalance of VEGF/Ang-2 ratio inhibits endothelial cell proliferation and prevents capillarization, the disarrangement of vascular formation, and reduced blood flow exacerbate hypoxia [85], further leading to impairment of AT insulin sensitivity.

In parallel, hypoxic condition has been regarded to be detrimental to adipocyte differentiation in hypertrophic AT. In both preadipocytes and adipocytes, hypoxia induces decreased acetylation level of histone H3 and H4 of peroxisome proliferator-activated receptor γ (PPARγ) promoter [81, 87]. However, varied degrees of hypoxia directly contribute to distinct effects on adipocyte differentiation. Improved adipogenesis after short period of hypoxic exposure was reported in previous studies. In one animal study, Sprague-Dawley rats were treated with 10 hypoxia cycles/h and 6 h/day, where one hypoxia cycle was defined as 240 s for 10% O_2_ and 120 s for 21% O_2_. After 6-week exposure, the hypoxia condition accelerated expressions of adipogenesis transcription factors CCAAT-enhancer-binding proteins α (C/EBPα), C/EBPβ, PPARγ, as well as insulin-like growth factor 1(IGF-1)/Akt signaling pathway in SAT [88]. Similar result was reported by another study in which C57Bl/6J mice were exposed to chronic hypoxia (8% O_2_). Decreased adipocyte size and improved adipose function were observed after 3-week treatment [87]. Since there is no standard duration of hypoxic exposure at present, further studies are needed to better clarify the range of hypoxia degree which could improve adipocyte differentiation.

### Accumulation of Extracellular Matrix

With the accumulation of lipid, increased deposition of collagen and reduced extracellular matrix (ECM) flexibility contribute to adipose dysfunction [89]. AT, as a connective tissue, contains a “collagen microenvironment”, which is composed of various types of collagen. Different types of collagen promote oriented differentiation of mesenchymal stem cells. Collagen I, III, and V are secreted mainly by fibroblasts, while collagen IV, VI, and XVIII (sparse) are secreted mainly by adipocytes [90, 91]. The former provides a “stiff” environment for a well-developed cytoskeleton in cells binding to these molecules. In contrast, the latter mediates changes in the cytoskeleton’s reorganization for adipocyte differentiation [90]. Physiologically, collagen is distributed in a balanced manner [90]. However, in obese state, collagen I and VI are the most common matrix prone to excessive synthesis [92]. One study showed negative correlation between total collagen content and SAS [93]. Another study confirmed this relationship that obese patients whose SAT contained greater collagen VI displayed small and medium adipocytes [94]. Likewise, VAT fibrosis level was negatively correlated with cell size and plasma triglyceride level [89]. Augmented collagen synthesis may be regarded as an adaptation mechanism in response to ameliorate adipocyte hypertrophy and limit adipose expansion.

Moreover, increased level of collagen in AT upregulates expression of FABP4 and partially upregulates PPAR-γ in adipocytes, resulting in alteration of metabolism of adipocytes [95]. These studies indicate that ECM redistribution in AT changed the morphology and metabolic function of adipocytes successively. Detailed response in cellular level will be discussed in the next section.

Collectively, adipocyte fibrosis seems to associate with limited adipocyte size and altered AT metabolism. Whether AT fibrosis serves as a more sensitive biomarker in reflection of adipose dysfunction still requires further investigations.

### Dysregulated Adipokine Release

Besides fat storage, AT also plays a key role in regulating systemic homeostasis through secretion of massive proteins. These peptide or non-peptide hormones are generally referred to as adipokines [61, 96]. During the progression of obesity, adipokine concentration and phenotype altered in response to sustained energy excess.

Leptin and adiponectin are well-studied adipokines that modulate appetite and energy metabolism respectively [96]. It was reported that SAS positively was associated with plasma leptin level in 83 over-weight subjects [9]. In addition, SAS was an independent predictor of plasma leptin level [97]. Similar study conducted in T2D patients suggested that within SAT, mRNA and protein levels of adipokines, such as leptin, serum amyloid A (SAA), and transmembrane 4 L six family member 1 (TM4SF1), were elevated in very large subcutaneous adipocytes compared with small adipocytes [60]. By contrast, patients characterized by adipocyte hypertrophy in both subcutaneous and omental AT had significantly lower plasma adiponectin level, though this impact was more evident in subcutaneous adipose compartment [75]. Also, in cell cultures, production of adiponectin is negatively linked to adipocyte size [98]. In general, hypertrophic adipocytes are accompanied by concomitant enrichment of proinflammatory, pro-diabetic adipokines, and decreased production of adipokines that exert beneficial effect on regional homeostasis.

### Impaired Glucose Metabolism

Although AT merely accounts for a small proportion of systemic insulin-induced glucose uptake, adipose GLUT4 specifically knockout mice displayed profound glucose intolerance, indicating that AT plays a crucial role in mediating glucose metabolism [99].

In normal state, the insulin receptor (IR) is located in caveolae. When insulin binds to the transmembrane receptor and activates protein tyrosine kinase, thus, intracellular docking proteins, including the IRS proteins, are recruited. Subsequently, PI3-kinase (PI3K) activation induces the activation of phosphoinositide-dependent kinase 1 (PDK1) [100], which then induces the activation of serine/threonine kinase (Akt)/ protein kinase B(PKB) and allows the translocation of glucose transporter type 4 (GLUT4). Importantly, within AT, insulin mainly induces glucose transport through intracellular localization glucose transporter GLUT4 [101]. Insulin increased exocytosis of GLUT4 through insulin-responsive vesicle pool. Then, increasing GLUT4 is transported to the plasma membrane and the rate of adipocyte glucose uptake was also markedly increased [101].

With the accumulation of adipose, the glucose uptake rate of hypertrophic adipocytes has changed significantly. Of note, previous study showed that, with the enlargement of adipocytes, their response to insulin in the instance of glucose utilization may decline persistently [102]. When patients underwent weight loss, their SAS was significantly decreased, with a parallel improvement of AT insulin sensitivity and plasma insulin level [102]. Likewise, SAS was proved negatively correlated with glucose uptake rate in SAT after adjustment for age, BMI, and sex [9, 103, 104]. One follow-up study reported no correlation observed between the measured GLUT4 storage vesicle (GSV) trafficking and adipocyte size isolated from different subjects [105]. Another study suggested that it might be inaccurate to compare the SAS from different individuals with distinct blood glucose level. This study sorted small and large SAT cells from the same individual and found that under the stimulation of insulin, an increase in GLUT4 located at the plasma membrane was only observed in small SAT cells, but not in large cells [106]. This interesting finding indicated that smaller adipocytes displayed increased glucose uptake rate while enlarged adipocytes associated closely with reduced insulin sensitivity in obese subjects.

In terms of VAS, no correlation was found between omental fat cell size and glucose uptake rate [9]. It was reported that insulin receptor binding affinity was greater in SAT than in VAT [107]. Thus, SAT may have a closer association to glucose uptake than VAT.

## Cellular Mechanisms Hypothesized to Regulate Hypertrophic Adipocytes

### Dysregulated Differentiation and Maturation of Preadipocytes in Obesity

The adipocytes originated from the potent fibroblast-like progenitor cells, such as MSCs. Generally, the process from MSCs to mature adipocytes involves two steps, whose impairment contributes to anomaly of adipocyte morphology.

In the first stage, adipocyte hypertrophy associates closely with inability to recruit and restrict MSC fate to adipogenic lineage during weight gain [108]. Previous studies have fully proved the canonical and uncanonical wingless-type mouse mammary tumor virus integration site family (WNT) signaling play critical role in regulating adipocyte commitment of precursor cells [109].

WNT1 inducible signaling pathway protein 2 (WISP2), an important WNT-associated molecular, has effect on both adipocyte commitment and differentiation. WISP2 commonly locates in cytosol. WISP2 presented in the cytosol formed a complex with PPARγ transcriptional activator zinc finger protein-423 (ZFP423). This complex is sequestered in the cytosol and the transcriptional activity of ZFP423 is repressed [110–112]. On the other side, extracellular WISP2 has been regarded as an atypical Wnt ligand [113, 114], which promotes the transcriptional activation of T cell factor/lymphoid enhancer-binding factor (Tcf/Lef) and induces β-catenin to the nucleus [115]. Interestingly, extracellular WISP2 (which also referred to as secreted WISP2 in some studies) promotes the proliferation of MSCs but also inhibits MSCs commitment of adipogenic lineage [112, 115]. Of note, WISP2 must be suppressed to ensure that MSCs would be restricted to adipogenic lineage [111]. Since previous study reported that WISP2 mRNA level positively correlated to SAS in 36 non-diabetic obese subjects [111], WISP2 serves as a potential target for improvement of adipocyte recruitment and differentiation.

Bone morphogenetic protein 4 (BMP4) is a ligand of the transforming growth factor-beta (TGF-β) superfamily, which recruits MSCs and commits them into adipogenic lineage. In general, BMP4 is secreted by mature adipocytes, acting as a feedback regulator to prevent abnormal adipocyte enlargement [116]. To promote adipocyte differentiation, BMP4 binds to BMP4 receptor and dissociates WISP2/ZFP423 complex by activating recombinant mothers against decapentaplegic homolog (SMAD)1/5/8 [116]. Therefore, ZFP423 enters the nucleus and initiates the transcription activity of PPARγ [111, 117]. Of note, increased BMP4 protein was found in hypertrophic adipocytes, which were regarded as “BMP4 resistance” [118]. Importantly, upregulated BMP4 inhibitor Gremlin-1 was observed in enlarged adipocytes. Reducing Gremlin-1 by specific antibodies significantly improved BMP4-driven adipocyte differentiation [113], suggesting that BMP-4 serves as a critical mediator in regulation of adipocyte differentiation.

In the second stage, preadipocytes gradually become mature adipocyte. PPARγ, a transcriptional factor, belongs to class I nuclear hormone receptor superfamily, serving as the central regulator of adipocyte differentiation [119]. When activated, PPARγ heterodimerizes with retinoid X receptor α (RXRα). These heterodimers bind to peroxisome proliferator hormone response elements (PPREs) [120] and control genes include fatty acid-binding protein (aP2), fatty acid transport protein (FATP) and fatty acid translocase (FAT/CD36), phosphoenolpyruvate carboxykinase (PEPCK), and acyl-CoA synthase, involving synthesis and lipolysis of triglyceride [120].

In addition, recent approaches emphasized several proteins which received less attention. EBF1, a transcription factor activated by C/EBPβ and C/EBPδ, could induce the activation of PPARγ. Both the activity and mRNA level of EBF1 were significantly lower in obese and hypertrophy subjects. In vitro, adipocyte volume negatively correlated with EBF1 gene expression in isolated adipocytes [13]. In addition, in line with PPARγ, C/EBPα binds on the promoter region of PPARγ and forms the self-reinforcing regulatory loop to further stimulate adipogenesis [121]. Lack of this transcriptional factor contributes to insulin resistance and obstruct WAT adipogenesis [122].

**Fig. 2:**
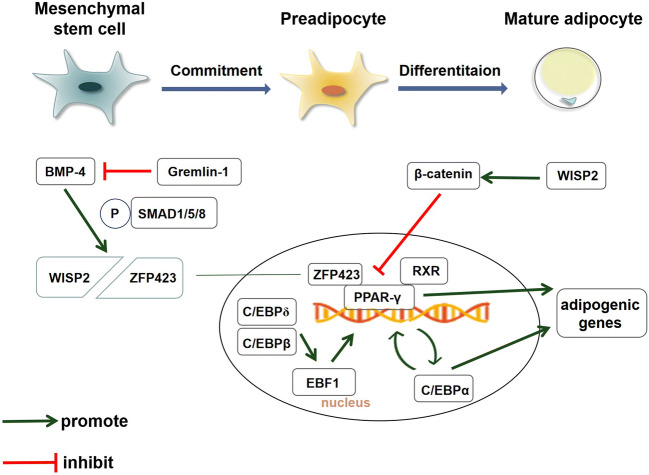
Molecular mechanism of adipogenesis. During the process of mesenchymal stem cells (MSCs) commitment to adipose lineage, WNT1 inducible signaling pathway protein 2(WISP2) presented in the cytosol repressed transcriptional activator zinc finger protein-423 (ZFP423) through formation of a WISP2/ZFP423 complex in cytosol. Bone morphogenetic protein 4 (BMP4) phosphorylates SMAD1/5/8 to dissociate WISP2/ZFP423 complex, making ZFP423 enter into the nucleus and further activates peroxisome proliferator-activated receptor γ (PPARγ). BMP4 inhibitor Gremlin-1 suppresses MSCs by inhibiting BMP4. Likewise, extracellular WISP2 inhibits PPARγ by activating β-catenin. After committed differentiation to adipogenic lineage, EBF transcription factor 1(EBF1), a transcription factor activated by CCAAT-enhancer-binding proteins (C/EBPβ) and C/EBPδ, induces the activation of PPARγ. C/EBPα binds on the promoter region of PPARγ and forms the self-reinforcing regulatory loop to further stimulate adipogenesis. Activated PPARγ heterodimerizes with retinoid X receptor α (RXRα) and promotes transcription of genes involved in adipocyte differentiation and lipid transport

Collectively, expressions of adipogenesis genes functioned as markers of how the adipose expanded (Fig. 2) [123]. Some clinical studies observed decreased expressions of pro-adipogenic genes in patients with insulin resistance or T2D compared with NGT subjects [35, 124, 125], suggesting that expressions of adipogenesis genes could reflect adipocyte turnover and can be used to evaluate the risk of developing T2D.

### Development of “Supersized” Lipid Droplet

There are various adaptive changes that occurred in adipocytes in response to excessive lipid accumulation, the most evident alteration of which is the enlargement of lipid droplets (LDs). [126]. Many factors contribute to the expansion of LDs. Abnormal composition of LD membrane is one of the factors that trigger LD enlargement. LDs consist of neutral lipid embraced by a layer of phospholipids which subbed with various integral and peripheral proteins [127]. Interrupting the synthesis of phospholipids is the main approach of dysregulating LD membrane formation. Importantly, recent approaches highlighted that aberrant proteins that control phospholipids lead to “supersized” LDs [128]. Seipin is an important integral membrane protein in the endoplasmic reticulum (ER) that concentrates at junctions with LDs and serves as an important regulator of lipid storage and lipid droplet budding. Deletion of seipin directly binds glycerol-3-phosphate acyltransferase (GPAT), probably manipulates acylation of glycerol-3-P and subsequent synthesis of triacylglycerol, delays lipid formation, and further induces anomalous LD morphology [129–131]. Yeast mutants with impaired phosphlipid phosphatidylcholine (PC) synthesis directly induced 50-times large “supersized” LDs [132]. Also, increased phosphatidic acid (PA) promotes augmented fusion of LDs, further contributing to the enlargement of LDs [132].

Besides phospholipid dysregulation, some embedded proteins, such as fat-specific protein of 27 kDa (Fsp27) and cell death-inducing DFF45-like effector a (Cidea), play critical roles in promoting lipid accumulation in supersized LDs. Both proteins are upregulated profoundly in mice with liver steatosis [133].

Apart from the intrinsic regulation, the architecture of adipocytes was also influenced by extracellular factors. Overnutrition induced by short-term overfeeding in mice rapidly causes onset of cellular insulin resistance in adipocytes [134], marked by impaired insulin signaling at the level of insulin receptor substrate (IRS)-1 [135, 136] and Akt [137]. Besides, IQ motif containing GTPase activating protein 1 (IQGAP-1) is reduced. Due to the interaction between IQGAP-1 and caveolae, it can promote the synthesis of cytoskeleton proteins such as F-actin and inhibit the expansion of lipid droplets [138]. In addition, caveolae mediates the redistribution of GLUT4. When F-actin increases and the activity of gelosin (endogenous actin degradation protein) is inhibited, caveolae’s motor ability is enhanced, which promotes the transport of GLUT4 to the cell membrane, thus improving insulin sensitivity [138].

ECM-adipocyte interaction is also an important mechanism in adipocyte remodeling. Limited adipocyte expansion in fibrotic AT is one of the most remarkable manifestations. Silver et al. shows that αβ integrin on adipocyte cell membrane is able to bind to collagen to activate Ras, mitogen-activated protein kinase kinase (MAPKK), and inhibitor of nuclear factor kappa-B kinase (IKK), then activating MAPK and NF-κB pathway [139]. Meanwhile, as a ubiquitously expressed basement membrane proteoglycan, collagen XVIII contains a unique frizzled-like domain that binds to Wnt10b. Adipocyte-collagen XVIII interaction activates the canonical Wnt pathway, and then interferes with the lipogenic phenotype of cells through GSK3β and TGF-β pathways [140, 141], despite the low concentration of collagen XVIII in AT. Additionally, the interaction between the partially degraded collagen and adipocytes reduces the assembly of actin and enlarges fat droplets [142]; thus, mechanisms underlying this alteration still require further investigation.

Notably, there are inconsistencies among studies concerning cellular structure. Hasson et al. show that in C57BL/6J mice treated with high-fat diet for 2 weeks, F-actin increased 4-fold in VAT compared with chow-fat group in response to the intact interaction between IQGAP1 and IRS-1. Reversed feeding correspondingly restored cell size, insulin response, expression of F-actin, and its regulatory proteins [143]. Although this study indicated the critical role of actin in adipocyte remodeling, the effect of cytoskeleton on the structure of adipocytes is quite contrary to the findings in other articles [138, 144]. Considering that studies listed above used different animal models and/or analyzed different AT depots, different signaling pathways (Akt pathway, Wnt-GSK3β pathway, etc.) were probably involved in reconstruction of adipocyte cytoskeleton. More in-depth studies are required to verify this depot specificity.

### Abnormal Osmolarity Sensors on Adipocytes

Generally, osmotic active compounds and extracellular tonicity exert great influence on cell volume. When encountered pathophysiological factors such as hypoxia or ischemia, cells induce generation of osmotic gradients by regulating plasma membrane ion channels and transporters [145]. Transient receptor potential cation channel subfamily V member 4 (TRPV4), an important swelling-activated channel, regulates osmotic pressure through mediation of Ca^2+^ influx [146]. When TRPV4 was knockout, the regulated volume decreases (RVD) were attenuated [147]. In adipocytes, TRPV4 knockout mice were protected from hypertrophy and insulin resistance [148], suggesting that TRPV4 plays an important role in promoting adipocyte hypertrophy. Another ion channel which influences cell size is swelling-activated voltage-regulated anion channel (VRAC). It was shown to initiate RVD via export of chloride ions (Cl^−^) [149]. Specifically, hypertrophic adipocytes displayed an increased “swell-activated” Cl^−^ current when compared with adipocytes with smaller size. Since activation of VRAC was due to swelling protein 1 (SWELL1) [150], SWELL1 emerges as one significant cell-autonomous sensor for adipocyte size regulation.

## Therapies Affecting Adipose Cell Size

### Bariatric Surgery

Bariatric surgery has been demonstrated to decrease fat mass and improve insulin sensitivity [151]. In recent years, increasing reports have highlighted that the change of adipocyte size might associate closely with metabolic homeostasis after Roux-en-Y gastric bypass (RYGB). One study tested SAT adipocyte volume of 217 obese and T2D women before surgery; they defined enlarged adipocytes as the threshold for patients whose HOMA-IR was in the first quartile. Notably, subjects whose SAS greater than the thresholds were less possible to improve their insulin resistance state 6 months after surgery [39]. Similar finding was observed in another cohort of 62 obese women before and 2 years after RYGB [38]. This study was challenged by one study which examined SAS of 61 obese patients with normal blood glucose level before and 2 years after RYGB. SAS was positively correlated to the improvement of HOMA-IR, while total fat mass and BMI level did not show correlation with Δinsulin or ΔHOMA-IR [47]. Variation of these studies could be explained by whether obese patients with T2D were recruited.

Interestingly, with weight loss after surgery, adipocytes tend to proliferate through hyperplasia rather than hypertrophy, leading to decrease in mean adipocyte size instead of reduced cell number. One study reported that patients who underwent bariatric surgery had stronger amplification ability and lower DNA damage in subcutaneous adipose-derived stromal cells (ASC), which makes adipocytes more likely to store energy in a proliferative rather than hypertrophic manner [152]. Another study highlighted that angiotensin II (Ang II), the main effector of RAS, represses differentiation of precursor cells, inhibits lipolysis, and causes adipocyte hypertrophy. When adipose-derived MSCs were exposed to Ang II for 13 days, lipid droplet formation together with adipose differentiation-specific marker was significantly decreased. Surprisingly, even patients who conducted RYGB 1 year ago had higher BMI levels compared with non-obese control subjects (29.1 ± 7.8 kg/m^2^ vs 26.9 ± 2.6 kg/m^2^), their plasma Ang II levels were significantly lower (52.1 ± 4.2 pg/ml vs 85.4 ± 12.4 pg/ml) [153].

Importantly, although bariatric surgery significantly reduces body weight and improves metabolic homeostasis in obese patients, some patients will undergo weight regain and are hard to maintain ideal body weight [154]. One study explored SAS and number in obese subjects who regained weight 2 to 5 years after bariatric surgery. Surprisingly, regardless of the increase in body weight, subcutaneous abdominal adipocyte number increased significantly while no change was found in adipocyte size compared with 2 years after surgery [155]. Also, another study found a prominent increase in adipocyte progenitors in both SAT and VAT 12 to 18 months after surgery [156]. Through the long-term observation, even if patients undergo weight regain, bariatric surgery could still initiate AT into a healthier metabolic phenotype by increasing adipocyte number instead of limiting adipocyte expansion.

### Calorie-Restricted Diet

Calorie restriction is also a common method besides conduction of bariatric surgery. Generally, both very low-calorie diets (VLCD) and low-calorie diets (LCD) contain essential nutrition but differ in the degree of calorie restriction [157]. Prior human study showed that after 4 weeks of VLCD, SAS was reduced by 11–12%. After 12 weeks of LCD, both abdominal and gluteal SAS were reduced by 15–20% [157]. Similarly, study in rodents suggested that when C57Bl/6 J mice underwent LCD (energy intake 70% of ad libitum intake) for 50 days, their VAS decreased significantly compared with control group [158].

In addition, some studies emphasized the change of adipocyte distribution rather than the absolute value of adipocyte diameter. Some studies in rodents found increased proportion of very small cells (< 20 μm) during LCD [98, 158]. In line with the animal study, one study recruited 57 subjects and they were randomly assigned to LCD group for 12 weeks or VLCD group for 5 weeks. Both groups displayed increased small adipocytes and decreased large adipocytes [159]. Intriguingly, one study showed that besides decreased adipocyte size, development of functional beige fat was also observed in lean C57BL/6 mice that underwent 4-week LCD [160]. These results indicated that the restriction on calorie intake, which induced negative energy balance, may lead to increased adipocyte progenitor commitment of new adipocytes and beige adipocytes, both of which suppress the unlimited expansion of adipocytes and exert favorable effects on metabolic homeostasis.

### Medicine or Bioactive Compounds

Thiazolidinediones, which abbreviated as TZDs, also known as glitazones, have been used for treatment of T2Ds. This drug exerts its effect primarily through activating adipogenesis transcription factor PPARγ. Specifically, TZDs induce receptors of fatty acids to bind to the RXR and further form a complex with PPRE. Then, genes in regulation of adipogenesis and lipid metabolism were augmented, with increasing fatty acids stored in adipocytes [161, 162].

One human study using abdominal SAT obtained from 12 overweight/obese non-diabetic, insulin-resistant individuals after 12 weeks of pioglitazone treatment. Subsequently, increased proportion of small adipocytes as well as a 25% increase in the absolute number of these cells were observed [163]. Therefore, TZD-induced promotion of adipogenesis significantly improved adipocyte hypertrophy, inflammation, and insulin sensitivity.

However, TZDs, such as troglitazone, pioglitazone, and rosiglitazone, have many side effects including idiosyncratic hepatotoxicity, fluid retention, and heart failure [161]. Thus, TZDs were withdrawn from clinical use as anti-diabetic drugs. Consequently, increasing new bioactive compounds have been explored to regulate adipogenesis in addition to TZDs. It was reported that berberine suppresses the mRNA and protein levels of adipogenesis-related transcription factors PPARc and C/EBPα and their upstream regulator, C/EBPβ [164]. In addition, Acid sphingomyelinase (Asm), a member of phospholipids, plays a critical role in regulating membrane composition via transformation of membrane lipid sphingomyelin to ceramide. Knockout of Asm mice displayed the absence of adipocyte hypertrophy and increased expression of genes related to brown adipocyte differentiation [165]. In the field of free fatty acid, when C57BL/6J mice were fed with high-fat (HF) diet supplemented with eicosapentaenoic acid (EPA) (45% of energy from fat; 36 g/kg EPA; HF + EPA) for 11 weeks, their body weight, total fat mass, and adipocyte size were remarkably reduced [166]. In spite of abundant bioactive compounds in regulation of adipogenesis, further studies are still required for clinical applications in ameliorating AT insulin resistance.

## Conclusion

Adipocyte size could reflect the progression of insulin resistance and T2D. In obesity, the function of adipose has been impaired, which could be reflected by several aspects including limited hyperplasic capacity of AT, inability to utilize glucose, and release of large amounts of fatty acids into circulation. We concluded the tight relationship between adipocyte size and metabolic dysregulation.

Most of the studies used absolute adipocyte size as an index for fat cell enlargement degree. Nevertheless, analysis of cell size distribution patterns seems to be another objective biomarker. Also, some researches concern the variance or minimal and maximal cell size. It is reasonable to take adipocyte variance into consideration for better understanding of cell size feature.

Up till now, large amounts of studies explored the relationship among adipocyte size, glucose or lipid metabolism, inflammation, and hypoxia in AT, since dynamic change of free fatty acids has been shown to be an important biomarker reflecting alterations of adipose function. With the accumulation of excessive energy intake, great amounts of fatty acids are released into circulation. It is thus logical to speculate that maybe enlargement of adipocytes was attributed to excessive or insufficient typical fatty acid.

Another factor that needs to be considered is gender impact on adipogenesis. We found most of the studies merely enrolled female subjects, especially studies related to bariatric surgery. Generally, female subjects have higher surgical intention. Also, it is hard to recruit male subjects in line with female ones when matched for age and BMI. However, one study highlighted that adipocyte distribution of different gender may vary profoundly as median adipocyte size in male individuals was much larger than that in female ones. Collectively, gender-specific threshold of hypertrophic adipocytes still requires further investigations.

## Abbreviations

T2D: Type 2 diabetes
WAT: White adipose tissue
AT: Adipose tissue
NAFLD: Non-alcoholic fatty liver disease
VAT: Visceral AT
SAT: Subcutaneous AT
VAS: Visceral adipocyte size
SAS: Subcutaneous adipocyte size
APC: Adipocyte precursor cells
GWAT: Gonadal white adipose tissue
IWAT: Inguinal white adipose tissue
ERα: Estrogen receptor alpha
ERβ: Estrogen receptor beta
HOMA-IR: Homeostasis model assessment of insulin resistance
IS: Insulin-sensitive
IR: Insulin resistance
IGT: Impaired glucose tolerance
NGT: Normal glucose tolerance
IFG: Impaired fasting glucose
AAS: Abdominal adipocyte size
FAS: Femoral adipocyte size
MS: Metabolic syndrome
ALT: Alanine aminotransferase
AST: Aspartate aminotransferase
PCOS: Polycystic ovary syndrome
TNF-α: Tumor necrosis factor α
IL-6: Interleukin-6
IL-1b: Interleukin-1b
CCR: Chemokine CC receptor
MCP-1: Monocyte chemoattractant protein 1
iNKT: Induced natural killer T
CRP: C-reactive protein
MSCs: Mesenchymal stem cells
EBF1: EBF transcription factor 1
HIF-1α: Hypoxia-inducible factor-1α
PPARγ: Peroxisome proliferator-activated receptor γ
IGF-1: Insulin-like growth factor 1
CEBPα: CCAAT-enhancer-binding proteins α
SAA: Serum amyloid A
TM4SF1: Transmembrane 4 L six family member 1
PI3K: PI3-kinase
PDK1: Phosphoinositide-dependent kinase 1
PKB: Protein kinase B
GLUT4: Glucose transporter type 4
GSV: GLUT4 storage vesicle
WNT: Wingless-type mouse mammary tumor virus integration site family
WISP2: WNT1 inducible signaling pathway protein 2
ZFP423: Zinc finger protein-423
Tcf/Lef: T cell factor/lymphoid enhancer-binding factor
BMP4: Bone morphogenetic protein 4
TGF-β: Transforming growth factor-beta
SMAD: Recombinant mothers against decapentaplegic homolog
RXRα: Retinoid X receptor α
PPREs: Peroxisome proliferator hormone response elements
aP2: Fatty acid-binding protein 2
FATP: Fatty acid transport protein
FAT/CD36: Fatty acid translocase
PEPCK: Phosphoenolpyruvate carboxykinase
LDs: Lipid droplets
GPAT: Glycerol-3-phosphate acyltransferase
PC: Phosphlipid phosphatidylcholine
PA: Phosphatidic acid
Fsp27: Fat-specific protein of 27 kDa
Cidea: Cell death-inducing DFF45-like effector a
IRS: Insulin receptor substrate
IQGAP-1: IQ motif containing GTPase activating protein 1
MAPKK: Mitogen-activated protein kinase kinase
IKK: Inhibitor of nuclear factor kappa-B kinase
TRPV4: Transient receptor potential cation channel subfamily V member 4
RVD: Regulated volume decreases
VRAC: Voltage-regulated anion channel
SWELL1: Swelling protein 1
RYGB: Roux-en-Y gastric bypass
ASC: Adipose-derived stromal cells
Ang II: Angiotensin II
VLCD: Very low-calorie diets
LCD: Low-calorie diets
Asm: Acid sphingomyelinase
EPA: Eicosapentaenoic acid
HF: High fat
